# Epstein–Barr Virus Gene BARF1 Expression is Regulated by the Epithelial Differentiation Factor ΔNp63α in Undifferentiated Nasopharyngeal Carcinoma

**DOI:** 10.3390/cancers10030076

**Published:** 2018-03-17

**Authors:** Eveline Hoebe, Coral Wille, Stacy Hagemeier, Shannon Kenney, Astrid Greijer, Jaap Middeldorp

**Affiliations:** 1 Department of Pathology, VU University Medical Center, 1081 HV Amsterdam, The Netherlands; hoebe@hotmail.com (E.H.); aegreijer@gmail.com (A.G.); 2 McArdle Laboratory for cancer research, Department of Oncology, University of Wisconsin School of Medicine and Public Health, Madison, WI 53705-2275, USA; coral.wille@gmail.com (C.W.); Stacy.hagemeier@gmail.com (S.H.); Kenney@oncology.wisc.edu (S.K.)

**Keywords:** EBV, NPC, transactivation, transcription, p63

## Abstract

Epstein–Barr Virus (EBV) BamHI-A rightward frame 1 (BARF1) protein is considered a viral oncogene in epithelial cells and has immune-modulating properties. During viral lytic replication BARF1 is expressed as an early gene, regulated by the immediate early EBV protein R. However, in viral latency BARF1 is exclusively expressed in epithelial tumors such as nasopharyngeal (NPC) and gastric carcinoma (GC) but not in lymphomas, indicating that activation of the BARF1 promoter is cell type specific. Undifferentiated NPC is characterized by high expression of ΔNp63 isoforms of the epithelial differentiation marker p63, a member of the p53 family of transcription factors. Transcription factor binding site analysis indicated potential p53 family binding sites within the BARF1 promoter region. This study investigated ability of various p53 family members to transactivate the BARF1 promoter. Using BARF1 promoter luciferase reporter constructs we demonstrate that only p63 isoform ΔNp63α is capable of transactivating the BARF1 promoter, but not the TAp63 isoforms, p53 or p73. Direct promoter binding of ΔNp63α was confirmed by Chromatin Immune Precipitation (ChIP) analysis. Deletion mutants of the BARF1 promoter revealed multiple ΔNp63 response elements to be responsible for BARF1 promoter transactivation. However, ΔNp63α alone was not sufficient to induce BARF1 in tumor cells harboring full EBV genomes, indicating that additional cofactors might be required for full BARF1 regulation. In conclusion, in EBV positive NPC and GC, BARF1 expression might be induced by the epithelial differentiation marker ΔNp63α, explaining BARF1 expression in the absence of lytic reactivation.

## 1. Introduction

Epstein–Barr virus (EBV) is associated with several human malignancies of B-cell origin, such as Burkitt’s lymphoma, Hodgkin’s disease and lymphoproliferative disorders in immune- compromised individuals, or epithelial origin such as nasopharyngeal carcinoma (NPC) and gastric carcinoma (GC) [1,2]. In EBV-related malignancies, several distinct gene expression patterns have been analyzed in detail (1). In addition to well-studied genes as Epstein-Barr nuclear antigen 1 (EBNA1) [3] and the latent membrane proteins 1 and 2 (LMP1 and LMP2) [2], EBV-associated carcinomas are characterized by the selective expression of BamHI-A rightward frame 1 (BARF1) [4,5,6].

The BARF1 gene encodes a 220 amino acid (aa) protein, of which the membrane associated leader peptide comprising the first 20 aa is cleaved off, leading to efficient secretion of hexameric BARF1 [7,8,9]. BARF1 may drive carcinogenesis by immortalizing and transforming epithelial cells and by blocking apoptosis, enabling cell survival [10]. In addition, secreted BARF1 has immune modulating properties, arresting growth and differentiation of mononuclear cells by functional inhibition of the macrophage colony stimulating factor (M-CSF) [11,12,13]. In B cells and lymphomas BARF1 is expressed in the early phase of the viral lytic replication cycle [10,14]. However in EBV positive carcinomas BARF1 expression is not restricted to the lytic phase, and can be detected in latently infected tumor cells. BARF1 protein is considered a viral oncogene in epithelial cells [5,6,15,16]. A detailed review discussing BARF1 oncogenic and immune modulating characteristics was recently published [17].

EBV positive carcinomas are predominantly undifferentiated and characterized by a high expression of p63 [18,19,20,21]. The transcription factor p63 is one of the key regulators of epithelial cell development and differentiation. In multilayered epithelial structures p63 protein diminishes from high levels in basal undifferentiated cells to low levels towards the differentiated cells at the surface [22]. p63 is the founding member of the family of transcription factors that also includes p53 and p73, sharing a common protein structure [23]. They consist of a central DNA-binding domain, a transactivation domain (TA), and a tetramerization domain. The strong homology among the different family members results in similar consensus sequences to transactivate target genes [24,25]. p63 and p73 contain an extended C-terminal coding region that undergoes complex alternative splicing [22]. The p63 gene uses two transcriptional start sites resulting in the expression of two major variants, called TA and ΔN which combined with alternative C-terminal splicing results in at least six transcripts: TAp63α, TAp63β, TAp63γ, ΔNp63α, ΔNp63β and ΔNp63γ. The existence of multiple p63 isoforms with various domains that can either activate or repress promoters adds to the functional complexity of the p63 gene [23,26,27]. Whether or not a gene is activated or repressed by the specific p63 isoforms is dependent on the cell type and the presence of cell specific cofactors [28,29,30].

Several cancers, including NPC, show an overexpression of ΔNp63. The p63 gene is rarely mutated in cancer, and several studies indicate that the (in) balance between isoforms rather than the reduction of one particular isoform might be important [31]. EBV might promote this imbalance by increasing the stability of p63 [19]. A number of EBV proteins induce expression of ΔNp63α such as latent membrane protein 2A (LMP2A) [32] and Epstein–Barr virus nuclear antigen leader protein (EBNA-LP, EBNA-5) [19], and EBV downregulates levels of cellular microRNA-203 whose target is p63 [33]. ΔNp63α upregulation reduces normal cell differentiation and might act to keep EBV in a latent state. Besides inhibition of differentiation, ΔNp63 is thought to play a role in cancer pathogenesis by driving cell cycle progression, inhibiting apoptosis and upregulating specific signaling pathways [18,31,34,35]. Expression of ΔNp63α was found to promote cell proliferation in both GC and NPC cells [21,24,36].

Thus far, there have been no suggestions in regard to why BARF1 is exclusively expressed in EBV positive epithelial malignancies and not in lymphomas, but the cellular background may point towards a role of dissimilar transcription factors. In this study the potential role of p53 family members in the epithelial restricted expression of EBV protein BARF1 was evaluated. Multiple potential p53 binding sites were found in the BARF1 promoter region. Specifically the ΔNp63α isoform was capable of inducing the BARF1 promoter. These findings suggest that BARF1 expression in undifferentiated carcinomas is mediated by the epithelial differentiation marker ΔNp63α.

## 2. Materials and Methods

### 2.1. Cell Culture

SNU-719, a naturally derived EBV-infected gastric carcinoma cell line was maintained in RPMI1640 medium [37]. AGS cells were maintained in Ham’s F-12 nutrient mixture medium. EBV infected 293 cells, EBV-positive AGS B95.8 (gifts from dr. Henri-Jaques Delecluse, German Cancer Research Center, Heidelberg, Germany) and R-stop EBV have been described previously [38,39] and were maintained under 100 µg/mL hygromycin B (Roche, Basel, Switzerland) selection. CNE-2 Akata cells (a gift from dr. Kwok-Wai Lo, Chinese University of Hong Kong, Hong Kong, China), a NPC cell line superinfected with the Akata strain of EBV, was maintained in RPMI1640 under 400 µg/mL G418 selection (Invitrogen, Carlsbad, CA, USA). C666.1, a NPC cell line consistently harboring EBV was cultured in DMEM in fibronectin-coated flasks (Sigma-Aldrich, Buchs, Switzerland). All media contained 10% FCS, 100 units/mL sodium penicillin, 100 µg/mL streptomycin sulphate and 2 mM L-glutamine.

### 2.2. Plasmids

pSG5 and pcDNA3.1 were obtained from Stratagene (La Jolla, CA, USA) and Invitrogen, respectively. The SG5-R expression vectors (kindly provided by Dr. S. Diane Hayward, John’s Hopkins University, Baltimore, MD, USA) were previously described [14,40]. Expression vectors for the various p53 family member isotypes were kindly provided by Dr. Elsa R. Flores (MD Anderson Cancer Center, Houston, TX, USA) and were described previously [41]. The BARF1 promoter region from −679 to the ATG start site (164367–165045) [GeneBank accession No. NC007605] was cloned into the pCpG.LUC, a CpG-free luciferase reporter vector kindly provided by M. Rehli [42], using forward primer with SpeI-site: CTGACTAGTCTCATCACGCAACACCCACTGTTT, and reverse primer with BglII-site: AATAGATCTGCTCTGGACTCTCCTCACCCAG [14]. Deletion mutants were constructed using forward primers closer to the ATG start site (Table 1). The plasmid was propagated in PIR expressing bacteria (Invitrogen). Plasmid DNA was purified on maxiprep columns according to the manufacturer’s protocol (Qiagen, Venlo, The Netherlands).

### 2.3. In Vitro DNA Methylation

The use of a CpG-free reporter construct enables to study the effect of promoter methylation without non-specific silencing due to methylation of the vector backbone. In vitro DNA methylation of the luciferase constructs was accomplished by CpG methylase (SssI methyltransferase; New England Biolabs, Ipswich, MA, USA), as recommended by the manufacturer. Completion of DNA methylation was confirmed by digestion with HpaII (New England Biolabs, Ipswich, MA, USA).

### 2.4. Luciferase Reporter Assays

Cells were seeded the day prior to transfection. Transfections were performed using Lipofectamine 2000 (Invitrogen) according to the manufacturer’s instructions, except that for the reporter assays the reagent: DNA ratio was 1.5 µL:0.5 µg in 100 µL Opti-MEM for 2 × 10^5^ cells plated in 1 mL medium in a 12-wells plate. Luciferase assays were performed 48 h after transfection by using extracts prepared by freeze-thawing the cell pellet in reporter lysis buffer according to the instructions of the manufacturer (Promega, Madison, WI, USA). Luciferase activity was assayed using the luciferase reporter assay system (Promega).

### 2.5. Chromatin Immunoprecipitation (ChIP) Assay

CNE-2 Akata and AGS B95.8 cells were transfected with expression vectors for TAp63α, ΔNp63α or an empty control expression vector using Lipofectamine 2000 (Invitrogen). Cells were first cross-linked in 1.5 mM EGS (ethylene glycolbis succinimidylsuccinate]) for 30 min at room temperature followed by cross-linking in fresh 1% paraformaldehyde for 10 min at RT. The cross-linking reaction was quenched using 125 mM glycine. Following IGEPAL (CA-630) mediated cell lysis and DNA fragmentation by sonication, DNA-protein complexes were immunoprecipitated with anti-p63 (NeoMarkers, Fremont, CA, USA), and control anti-IgG (Santa Cruz Biotechnology, Santa Cruz, CA, USA) antibodies. Protein-DNA cross-linking was reversed at 65 °C overnight, and DNA was purified using the Qiagen gel extraction kit. The presence of BARF1 promoter DNA fragments in each precipitate was detected using PCR using forward primer: GGCCCTGAACATGAGGTAGC and reverse: GCCAACAGGAGGAGCTGAGC, and those for E-cadherin were forward: CATGGCTCACACCTGAAATCC and reverse: AGTACAGGTGCACACCACCA and JAG-1 forward: ACCTTTCACCATTCCCCTAC and reverse: GCCCAAGGACAAAATAGCCA as previously used by Testoni et al. [43]. Primers for the negative control β2M were forward: AGGGCTGGGCATAAAAGTCA and reverse: GCCTCACCACCAACTTCATC.

### 2.6. Quantitative RT-PCR

Cells were plated in 6 well plates and expression vectors for TAp63α, ΔNp63α or an empty control expression vector using Lipofectamine 2000 (Invitrogen). After 48 hours cells were harvested in 1 mL Trizol (Invitrogen). Guanidinium isothiocyanate-phenol-chloroform extraction was performed to isolate total cellular RNA, followed by DNase (Promega) treatment and ethanol-precipitation. cDNA was synthesized using AMV Reverse Transcriptase (Promega) and sequence specific primers: BARF1 forward: GCCTCTAACGCTGTCTGTCC and reverse: GAGAGGCTCCCATCCTTTTC, U1A forward: CAGTATGCCAAGACCGACTCAGA and reverse: GGCCCGGCATGTGGTGCATAA. RT-PCR was performed with SybrGreen (Roche) and afore mentioned primers using the LightCycler^®^ 480 system (Roche) After quantification to known concentration of the corresponding gene constructs, values were normalized to U1A.

### 2.7. SDS-Page and Western Blot

Lysates from luciferase assay and ChIP were diluted in 2× loading buffer (Biorad, Hercules, CA, USA) with β-mercaptoethanol, denatured for 5 min at 95 °C and separated on a 10% SDS-page gel. After transferring to Hybond ECL nitrocellulose membrane (GE Healthcare, Little Chalfont, UK), the membrane was blocked in PBS with 0.05% Tween-20 (PBST) containing 3% non-fat dried milk for 1 h at RT, after which anti-p63 (Santa Cruz Biotechnology) was incubated for 2 hours at RT in PBST containing 5% BSA. After incubation with peroxidase labeled secondary antibody rabbit anti-mouse (Dako, Glostrup, Denmark) bands were visualized with ECL+ (GE Healthcare). Positive control lysates for endogenous p63 were obtained by pBABE-LIC-ΔNp63α transfected 293T cells. Briefly, ΔNp63α was PCR amplified from pcDNA3.1-ΔNp63α and cloned into pBABE-LIC vector (obtained from Dr. Priyamvada Rai, University of Miami, USA) as described [44].

## 3. Results

### 3.1. Potential p53 Family Response Elements Found in the BARF1 Promoter

**Figure 1:**
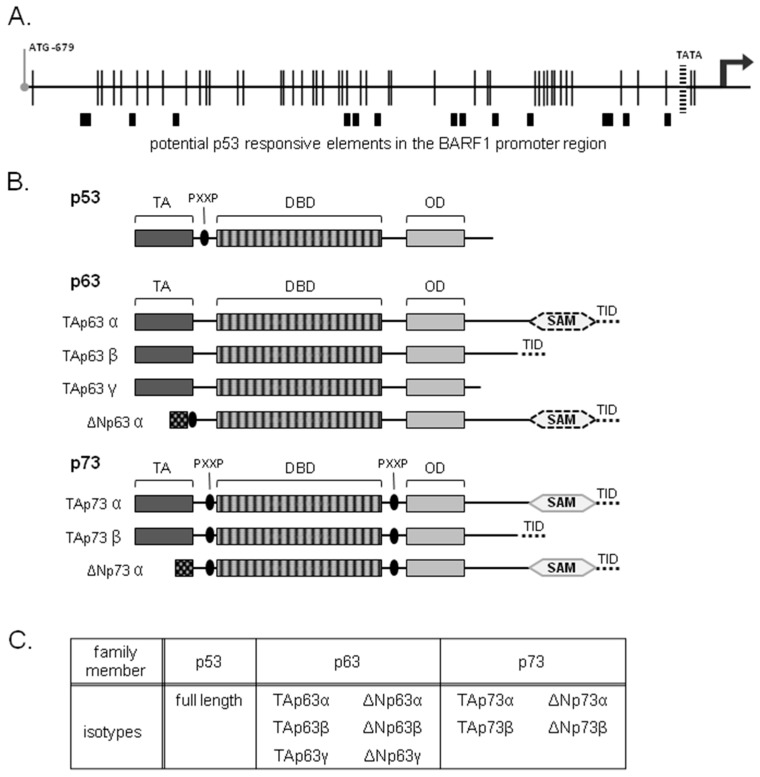
The p53 family of transcription factors and potential binding sites in the BARF1 promoter region. (**A**) Schematic overview of p53 family proteins. Transactivating domain (TA); DNA binding domain (DBD); oligomerization domain (OD); sterile alpha motif (SAM); transactivation inhibitory domain (TID); PXXP motif; (**B**) Potential p53 family member binding sites in the BARF1 promoter region. Black vertical lines represent methylation sites. A BARF1 promoter reporter construct was created by inserting the promoter sequence, up to −679 nucleotides from the ATG start site upstream of the luciferase gene in a CpG-free reporter construct; (**C**) overview of expression vectors containing the various isoforms of the different p53 family members used in reporter assays.

EBV protein BARF1 is selectively transcribed in undifferentiated NPC and GC associating with viral latency, but not in EBV associated B cell malignancies unless the lytic cycle is activated. To analyze which cell type specific transcription factor could potentially transactivate the BARF1 promoter, a transcription factor search was performed on the BARF1 promoter region from −680 bp through the promoter area of the BARF1 gene to the ATG start site, as defined recently [14]. Using Match Matrix Search for Transcription Factor Binding Sites [45] a list of potential transcription factors was generated which was evaluated for cell type specific factors. The search revealed that the BARF1 promoter harbors several binding sites for p53 transcription factor family members (Figure 1A).

### 3.2. ΔNp63α, But No Other p53 Family Members, Transactivates BARF1 Promoter in Luciferase Reporter Assays

The epithelial differentiation marker ΔNp63α, a member of the p53 family, is upregulated by EBV and shows elevated levels in NPC and GC [18,19,20,21]. Individual p53 family members (Figure 1B) were cloned and expressed in epithelial cells co-transfected with various BARF1 promoter-reporter constructs. To study the transactivation potential of the p53 family members on BARF1 activation, the BARF1 promoter sequence, up to −679 nucleotides from the ATG start site, was inserted upstream of the luciferase gene in a pCpG-free reporter construct. The reporter construct was co-transfected with individual expression vectors containing the various isoforms of the different p53 family members (Figure 1C) in AGS cells, an EBV negative GC cell line, and SNU-719 cells, a naturally derived EBV-infected GC cell line. Induction of luciferase activity was evaluated 48 hours after transfection. Expression of comparable levels of p53 and p73 isoforms showed no induction of luciferase activity (Supplementary Figure S1).

**Figure 2:**
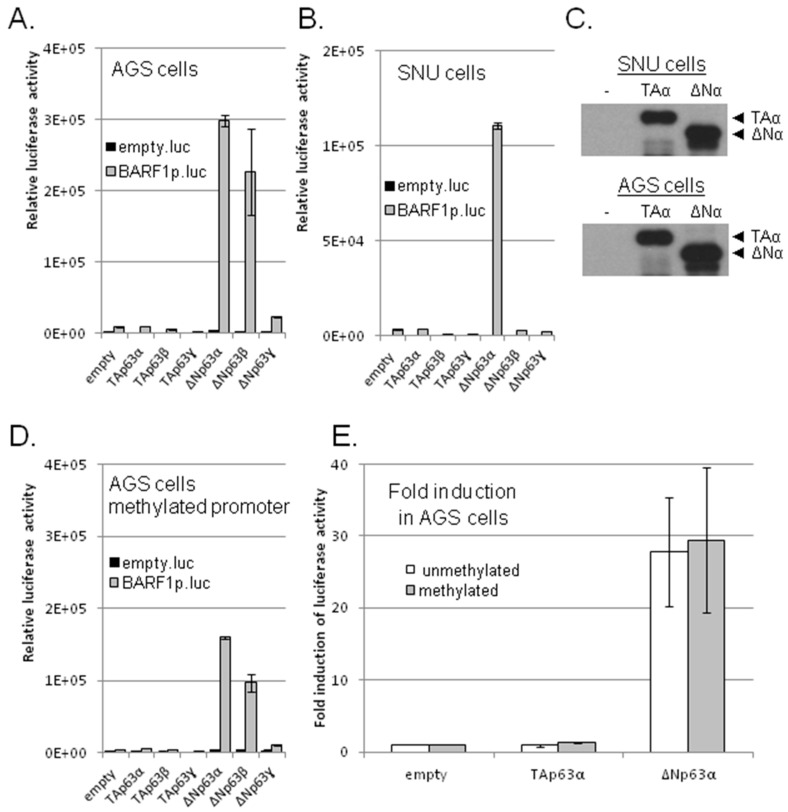
The BARF1 promoter reporter construct is transactivated by the ΔNp63α isoform. (**A**) Representative graphs of a cotransfection of the reporter construct with individual p63 isoforms in AGS and (**B**) SNU-719 cells. Only the ΔNp63α isoform shows transcriptional activity in both cell lines; (**C**) Representative SDS-page Westernblot of both the TAp63α and ΔNp63α isoform demonstrates comparable protein levels; (**D**) Methylation of the BARF1 promoter reduces relative luciferase activity induced by ΔNp63α, representative graph; (**E**) Luciferase activity is 28 fold upregulated by ΔNp63α and not by TAp63α (*n* = 7). Fold induction induced by ΔNp63α compared with constitutive activity of the methylated promoter is unchanged (29 fold) (*n* = 3).

However, among the p63 family isoforms, particularly the ΔNp63α isoform showed specific transactivation of the BARF1 promoter in both AGS and SNU-719 cells (Figure 2A,B). Next to ΔNp63α, in AGS cells the ΔNp63β isoform also induced luciferase activity. Expression levels of individual p63 isoform proteins were similar as analyzed on Western blot (Figure 2C). The TAp63 isoforms, considered to be the transcriptionally active p63 form with a proper transactivation domain, did not induce luciferase activity. The difference between the TAp63α and ΔNp63α isoforms lies in absence of the TA1 transactivating domain and the gain of a putative TA2 transactivating domain with different characteristics in ΔNp63α.

The EBV episome is highly methylated in latent carcinoma [46] and previous research by our group demonstrated a high level of CpG methylation of the BARF1 promoter region [14]. To investigate to which level BARF1 promoter transactivation by ΔNp63α is affected by methylation, the pCpG luciferase construct containing the BARF1 promoter was methylated in vitro using methyltransferase. In transfected AGS cells, both the basal and the ΔNp63α induced activity of the BARF1 promoter-reporter construct was only slightly weaker in the methylated construct (Figure 2D), leaving the fold induction by ΔNp63α mostly unaffected by methylation (Figure 2E). Repeated experiments showed that the TAp63α isoform has no effect on the BARF1 promoter while the ΔNp63α isoform gave rise to an average 28-fold induction irrespective of the promoter methylation status (Figure 2E).

### 3.3. ΔNp63α Is Complexed with the BARF1 Promoter In Vivo

**Figure 3:**
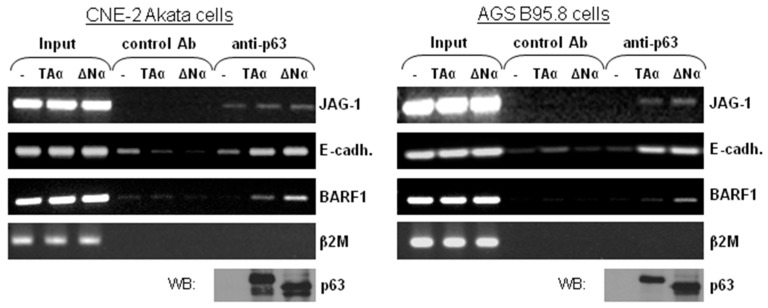
ΔNp63α directly complexes with the BARF1 promoter. Chromatin Immune Precipitation (ChIP) assays were performed using extracts from CNE-2 Akata and AGS B95.8 cells transfected with TAp63α, ΔNp63α or a control expression vector (−). p63 was immuneprecipitated by a control antibody or a non-isotype specific anti-p63 antibody, and co-immuneprecipitated DNA was PCR amplified. JAG-1 and E-cadherin served as positive controls for p63 binding and beta-2 microglobulin (β2M) served as negative control. The band in lane 9, indicates that the promoter region DNA is precipitated with ΔNp63α.

The ΔNp63α-mediated activation of the BARF1 promoter could either involve direct binding via the DNA-binding domain, or via the previously defined interaction of ΔNp63α with a CCAAT binding factor [35,47]. Evaluation of the BARF1 promoter region showed us that no CCAAT box is present in the sequence. To determine if ΔNp63α is complexed with the BARF1 promoter region in EBV harboring cells, Chromatin Immune Precipitation (ChIP) assays were performed. Gastric carcinoma AGS B95.8 cells and nasopharyngeal carcinoma CNE-2 Akata cells, both containing EBV episomes, were transfected with TAp63α or ΔNp63α expression vectors or a control vector, treated with a crosslinking agent and lysed. Both p63 isoforms were precipitated by anti-p63 antibody, which binds all isotypes. Binding of p63 to the known JAG-1 p63 intronic binding site and E-cadherin promoter served as positive controls (44). Cross-linked DNA was PCR amplified using primers for the BARF1 promoter, the E-cadherin promoter and the JAG-1 intronic binding site. The ChIP assay showed that p63 binds to the BARF1 promoter region or in its immediate proximity as well as to the E-cadherin and JAG-1 promoters (Figure 3). Since the p63 antibody does not differentiate between isoforms, the bands visible in TAp63α transfected cells were probably due to non-activating binding of TAp63α.

### 3.4. Multiple p63 Response Elements Are Responsible for BARF1 Promoter Transactivation

**Figure 4:**
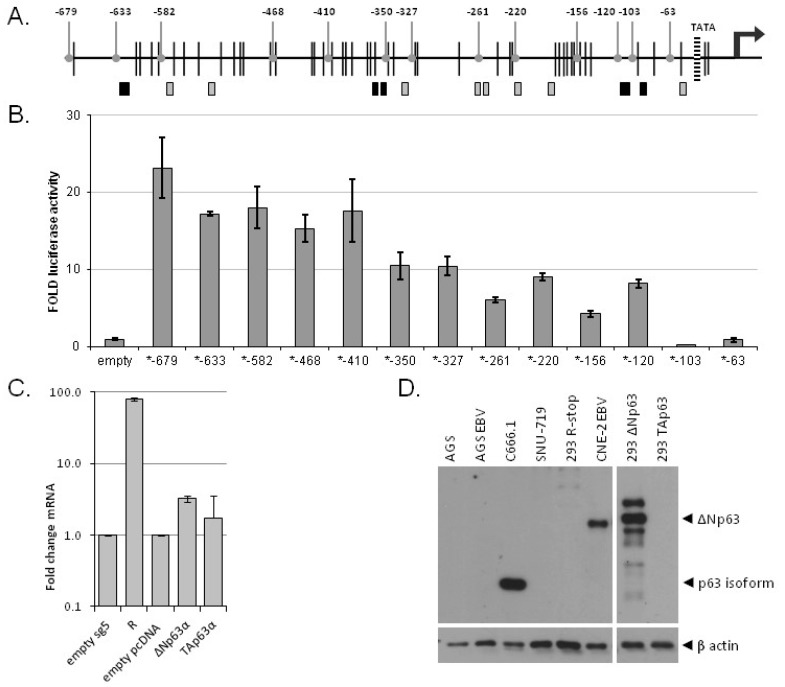
Multiple p63 response elements are responsible for BARF1 promoter transactivation. (**A**) Potential p53 family responsive elements are depicted on the BARF1 promoter region (boxes). Black vertical lines represent methylation sites. Rounded grey indicators point to the deletion mutants made from the BARF1 reporter construct, shortening the BARF1 promoter sequence from the original −679 to −63 relative to the ATG start site; (**B**) AGS cells were transfected with the deletion mutant luciferase constructs and with or without ΔNp63α expression vector. The ΔNp63α induced luciferase activity (fold) was measured 48 hours after transfection. A representative experiment is shown; (**C**) 293RKO cells demonstrate that, unlike R, ΔNp63α has only minor transactivating activity (3 fold) of BARF1 in the context of the intact viral genome; (**D**) Endogenous p63 levels as detected by Western blot, HEK293 cells stably expressing either TAp63 or ΔNp63 were used as positive control.

To identify the transcription factor binding site responsible for BARF1 promoter activation by ΔNp63α, deletion mutants of the reporter construct were made, shortening the BARF1 promoter sequence starting from the original 679 nucleic acids upstream of the ATG start site (ATG-679) to ATG-63 in small steps (Figure 4A). After removal of the region between ATG-679 and −410 an almost two fold drop in luciferase activity was noticed (Figure 4B). Luciferase activity was completely lost when only 103 nucleotides of the BARF1 promoter remained, indicating that multiple ΔNp63α response elements exist on the BARF1 promoter in close proximity to the TATA-box. This is in agreement with the distribution of the potential p53 family member binding sites indicated as boxes in Figure 4A. The black boxes indicate binding sites likely to be most important for ΔNp63α BARF1 transactivation.

### 3.5. ΔNp63α Alone Is Not Sufficient to Induce BARF1 in Context of the Viral Genome

To obtain evidence that ΔNp63α is capable of transactivating BARF1 expression in the context of EBV infection, two different EBV positive epithelial cells were transfected with ΔNp63α, TAp63α or empty control vectors. As a positive control, an R expression was used to induce BARF1 mRNA as described before [14]. Quantitative RT-PCR of BARF1 mRNA was used to analyze whether BARF1 mRNA could be induced in context of the intact wild type viral genome. At 48h hours after transfection with the ΔNp63α expression vector neither AGS B95.8 nor C666.1 cells showed induction of BARF1 mRNA above the natural basal levels in each cell line (Supplementary Figure S2). Similar studies were done in 293-cells carrying recombinant-EBV. In order to prevent background levels of BARF1 transactivation by the lytic transactivator R, 293HEK cells carrying recombinant R-stop EBV (293RKO) were used [14,39]. Only in these cells a minor induction (3 fold) of BARF1 mRNA by ΔNp63α could be detected (Figure 4C). Previous studies showed low p63 levels in 293HEK cells as compared to C666.1 [19,32]. Possibly the steady-state p63 levels in the AGS B95.8, CNE-2 Akata, and C666.1 cell lines are at maximal levels above which additional BARF1 transactivation cannot be detected. However, we were not able to detect endogenous p63 using the available antibody on Western blot (Figure 4D).

## 4. Discussion

In vivo, the EBV-encoded BARF1 gene is exclusively transcribed in EBV-positive undifferentiated epithelial malignancies, like NPC and GC. In these tumors EBV remains in a latent state and the regulation of BARF1 expression remains undefined [5]. We recently demonstrated that BARF1 expression is induced by the immediate-early transactivator R during early stages of lytic EBV reactivation in both epithelial and B-cells [14]. Here we identified the differentiation specific transcription factor, ΔNp63α as a putative regulator of constitutive latent BARF1 expression in the epithelial background.

EBV associated nasopharyngeal and gastric cancers are characterized by high levels of the p53 family member ΔNp63α [18,19,20,21] and ΔNp63α is considered essential for cell cycle progression [34]. Analysis for potential transcription factor binding sites revealed that the BARF1 promoter region has multiple potential binding sites for the p53 family of transcription factors. Using promoter reporter constructs we showed that ΔNp63α is capable of transactivating the BARF1 promoter up to 30 fold where other p53 family members have no effect. Chromatin immunoprecipitation with cells harboring the full EBV genome demonstrated that ΔNp63α binds to the BARF1 promoter region or in its immediate proximity.

The protein p63 is essential for epithelial cell development and differentiation. Previous studies have shown that ΔNp63 is responsible for maintaining the proliferative potential of basal epithelial cells, while the interplay of TAp63 with ΔNp63α would facilitate epithelial differentiation [25]. EBV positive carcinomas are predominantly undifferentiated and have high p63 expression levels [18,19,20,21,36]. In latent B-cells, which are the natural reservoir for EBV, the dominant p63 isoform in B-cells is the TAp63α isoform and not ΔNp63α. BARF1 expression is linked to viral latency in epithelial cells, but not in B lymphocytes and therefore ΔNp63α may be one of the responsible cell type specific transcription factors regulating latent BARF1 gene expression.

A number of viruses utilize the epithelial differentiation process for productive viral replication and a tight control of p63 expression is essential for the survival of these viruses. Kaposi’s sarcoma-associated herpesvirus (KSHV) [48], human papilloma virus (HPV) [49] and EBV [50,51,52] all produce their viral progeny in differentiated cells albeit major differences exist in execution of this viral reproductive route. The HPV productive process is studied the most intensively using organotypic raft cultures, which duplicate epithelial differentiation in vitro, revealing that regulation of p63 is essential for HPV replication [53,54]. ΔNp63α is affected by multiple HPV proteins such as E6, which is thought to influence ΔNp63α function by enhancing its activity [55], and E5 and E7 that increase ΔNp63α levels by downregulation of skin-specific cellular microRNA203 (miR-203) expression [56,57]. High expression of ΔNp63α is also related with HPV carcinogenesis, accentuated by the finding that it correlates with a higher rate of progression of cervical low grade lesions and carcinoma [58].

Likewise in the EBV life cycle, the differentiation status of the host cell is essential in determining whether new viral progeny will be made, or whether the virus remains in a latent state and thereby hiding from the immune system. A differentiation responsive element can be found in the promoter of the lytic switch protein BZLF1 (Z, Zta, ZEBRA) [59], turning on lytic replication when the host cell initiates differentiation. Prevention of normal differentiation by elevated ΔNp63α levels might act to keep EBV in a latent state [50,51,52] and multiple EBV proteins have been found to influence p63. Early after infection Epstein–Barr virus nuclear antigen 5 (EBNA5, EBNA-LP) interacts and stabilizes p63, reducing normal differentiation and bringing EBV to a immunogenic-safe latent state [19,60]. The stabilizing function of EBNA5 on p63 is later taken over by other latent EBV proteins. MicroRNA profiling of EBV infected epithelial cells and NPC tissues demonstrated that, similar to HPV infection, EBV causes downregulation of cellular miR-203 levels [33]. LMP1 is responsible for this miR-203 downregulation, and it is plausible that this will contribute to higher levels of its target ΔNp63α. LMP2A physically associates with ΔNp63α, increasing protein level and stability [32]. Both LMP1 and LMP2A have been shown to increase β-catenin transcriptional activity in epithelial cells [61,62] and shortly hereafter β-catenin proved to be a key regulator of ΔNp63α expression [63]. These findings suggest that LMP1 and LMP2A act via the β-catenin pathway involved in activation of ΔNp63α transcription.

The differentiation status of EBV positive cells is also influenced by cyclin D1 [64], whose expression is activated by the earlier mentioned β-catenin pathway [65], as well as by BARF1 [66]. In addition, microRNA-34a/c targeting cyclin D1, is downregulated by p63. Although ΔNp63α and cyclin D1 co-localize in the basal layer and family member p73 interacts with certain cyclins [67], no direct interaction has been established between p63 and cyclin D. The exact mechanism leading to inhibition of differentiation might differ among cell types [14,68,69,70].

Overexpression of ΔNp63α was not sufficient to activate BARF1 expression in the context of the intact viral genome, although ΔNp63α was capable of transactivating the BARF1 promoter reporter construct. The inability of solely ΔNp63α to activate BARF1 expression indicates that specific chromatin modeling as well as other cofactors might be required for full BARF1 regulation. Previous research by our group indicated that the BARF1 promoter is almost completely methylated both in NPC and Burkitt’s lymphoma cells [14,46,71]. Methylation of the BARF1 promoter region does not seem to have a direct effect on the transactivating capability of ΔNp63α. In addition to DNA methylation, histone modification of the promoter will also regulate the accessibility to transcription factors, and this may be cell type dependent [19,32].

The inability of ΔNp63α to upregulate BARF1 expression can also be explained if the maximum BARF1 promoter transactivating capacity of ΔNp63α was already expressed. The cell lines tested are all undifferentiated (293, AGS, C666.1) or poorly differentiated (CNE-2) expressing at base line already p63 [29], leaving the possibility that the BARF1 mRNA expression induced by ΔNp63α in these cells is already at its peak. However, these endogenous p63 levels were not detected with Western blot. Future studies might elucidate the identity and importance of the unknown host or viral factors in BARF1 gene regulation.

## 5. Conclusions

In conclusion, this study provides the first indication that expression of the BARF1 protein during viral latency in epithelial cells is potentially regulated by the epithelial differentiation factor ΔNp63α.

## Figures and Tables

**Table 1:** Primers for BamH1-A rightward frame-1 (BARF1) promotor reporter vector. Forward primers used to construct deletion mutants of the BARF1 promoter luciferase reporter vector starting 679 nucleotides upstream the ATG start site. Underlined: SpeI restriction site.

ATG-n	Forward primers
679	CTGACTAGTCTCATCACGCAACACCCACTGTTT
633	CTGACTAGTAAGTCAGTCAGGCTGGCCAGG
582	CTGACTAGTGATCTTGGCATGCCGCCCAGC
468	CTGACTAGTACCGCAAACACCACTGTGTAGC
410	CTGACTAGTGGTCGTTGTACACTGCGCGCAG
350	CTGACTAGTCGATGTCGGCTGTCCTGCAGG
327	CTGACTAGTAGCTCCGCGTACAGCTTCCTATCC
261	CTGACTAGTGGCAAAGGCAGGTCTTTCTCATCC
220	CTGACTAGTCATGGCCCTGAACATGAGGTAGC
156	CTGACTAGTCACGCCTCGACCGGGGTC
120	CTGACTAGTGTAGACTTGGCTGGCCTCATGG
103	CTGACTAGTCATGGTCTCGTCAGGCCAGC
63	CTGACTAGTTGATAAAATGGGCGTGGCAG

## Supplementary Materials

The following are available online at http://www.mdpi.com/2072-6694/10/3/76/s1, Figure S1: 1-ΔNp63α_transactivates_BARF1-Hoebe, Figure S2: 2-ΔNp63α_transactivates_BARF1-Hoebe.
