# Supplementary material for: Epstein–Barr Virus Gene BARF1 Expression is Regulated by the Epithelial Differentiation Factor ΔNp63α in Undifferentiated Nasopharyngeal Carcinoma
Source: Cancers (Basel). 2018 Mar 17;10(3):76. doi: 10.3390/cancers10030076 (PMC5876651; doi:10.3390/cancers10030076)
Supplement: Supplementary file 1 [file cancers-10-00076-s001.pdf]

Supplementary Materials: Epstein-Barr virus gene BARF1 expression is regulated by the epithelial differentiation factor ΔNp63α in undifferentiated nasopharyngeal carcinoma.

Eveline Hoebe, Coral Wille, Stacy Hagemeier, Shannon Kenney, Astrid Greijer and Jaap Middeldorp

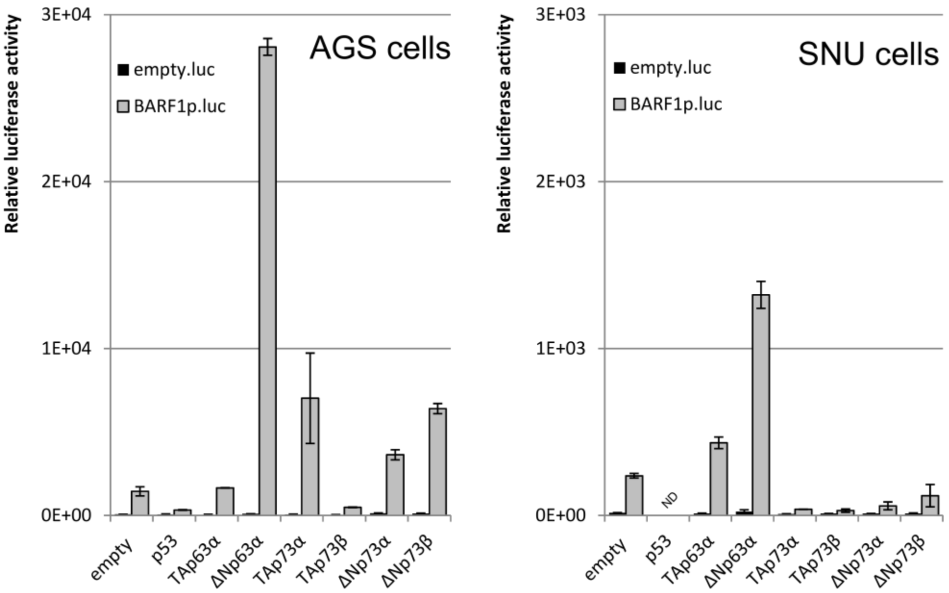

Figure S1. 1-ΔNp63α_transactivates_BARF1-Hoebe-2013

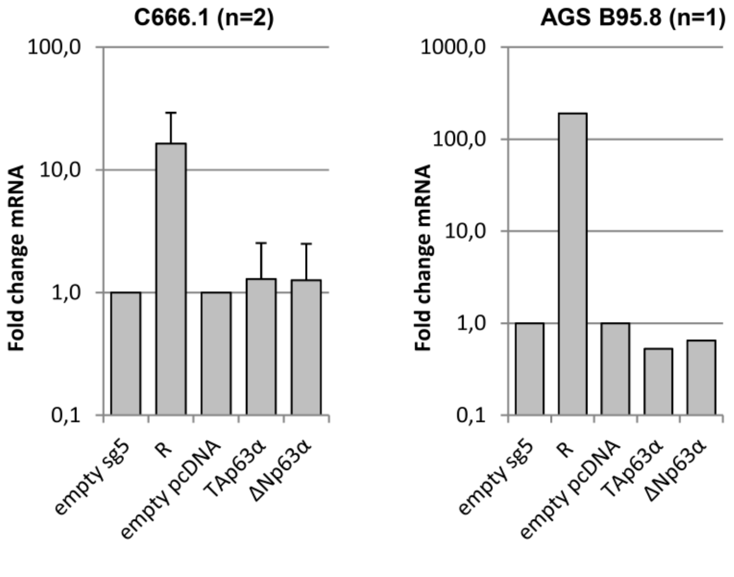

Figure S2. 2-ΔNp63α_transactivates_BARF1-Hoebe-2013

© 2018 by the authors; licensee MDPI, Basel, Switzerland. This article is an open access article distributed under the terms and conditions of the Creative Commons by Attribution (CC-BY) license (http://creativecommons.org/licenses/by/4.0/)
